# A review of environmental factors implicated in human developmental dysplasia of the hip

**DOI:** 10.1007/s11832-014-0615-y

**Published:** 2014-10-25

**Authors:** Amanda M. L. Rhodes, Nicholas M. P. Clarke

**Affiliations:** 1 Trauma and Orthopaedics, University Hospital Southampton NHS Foundation Trust, Southampton, England, UK; 2 MP817, Department of Child Health, Southampton General Hospital, Tremona Road, Southampton, SO16 6YD England, UK

**Keywords:** Developmental dysplasia of the hip (DDH), Nutrition, Environmental, Hormonal, Epidemiological

## Abstract

**Purpose:**

Developmental dysplasia of the hip (DDH) is common, and the term encompasses a spectrum of anatomical abnormalities of the hip in which the femoral head displaces from the acetabulum. These abnormalities may be congenital or develop during infancy and/or childhood. Neither the prenatal and postnatal factors that predispose to hip instability nor the determinants of its resolution or persistence are well characterised. A multifactorial pathogenesis of DDH is commonly accepted and identified risk factors include a family history, being first born, breech presentation, female gender, high birth weight and oligohydramnios 1. Further to genetic factors, a number of nutritional, hormonal and mechanical influences on ligament laxity have been hypothesised.

**Methods:**

A comprehensive search was conducted using NICE Healthcare Databases Advanced Search and Google Scholar engines, and the terms “nutrition”, “environmental”, “risk factors”, “CDH” and “DDH”. Wherever possible, evidence from randomised controlled trials, systematic reviews and expert review articles published in the medical and veterinary literature was considered.

**Results:**

The relationship between a number of hormones and biochemical markers of nutritional status and the development of DDH has been repeatedly hypothesised upon in the last 45 years. Of those most frequently cited are calcium, vitamins C and D, and relaxin hormone. The evidence for these potential risk factors is provided mainly by canine studies, with a paucity of consistent or strong evidence in humans.

**Conclusions:**

DDH is common and remains a leading cause of hip osteoarthritis in young adults. Neonatal clinical screening programmes for this condition have been in practice since the 1950s, albeit with varying levels of sensitivity. This review summarises current understanding of some of the most frequently cited nongenetic hypothesised risk factors, the significance of which remain to be determined.

## Clinical relevance

Improved characterisation of a number of possible environmental risk factors would advance accurate identification of not only those at risk of DDH, but also differentiation between infants with hip instability that is like to resolve and those in which it may persist. The establishment of a large clinical inception cohort study would provide a valuable data set, from which hypothesis generation for further focussed studies may subsequently be developed.

## Environmental factors implicated in human developmental dysplasia of the hip

Developmental dysplasia of the hip (DDH) is common and remains a leading cause of hip osteoarthritis in young adults. Predating Ortolani’s well-recognised work in the 1900s, the first published report of clinical hip instability is believed to have been in 1879 [1]. That DDH continues to be regarded as “mysterious, protean and unresolved” [2] pertains to its intriguing complexity.

The term DDH encompasses a spectrum of anatomical abnormalities of the hip, which may be either congenital or develop during infancy and/or childhood. A multifactorial pathogenesis of DDH is commonly accepted. A broad range of risk factors is quoted in textbooks and journals. These include being first born, female gender, high birth weight, oligohydramnios, talipes (calcaneo valgus or equino varus), a family history and breech presentation. Of these, only the latter two are recommended in national screening programme guidelines to identify at-risk babies for secondary screening [3].

Neonatal clinical screening programmes for DDH have been in practice since the 1950s, accompanied by considerable debate as to their effectiveness and impact on functional outcome. The importance of early diagnosis, however, is universally acknowledged: the earlier the detection, the simpler and more effective the treatment. An increasing burden on secondary ultrasound screening services at a rate of 5 % year on year [4] propels the need for improved characterisation of risk factors predisposing to DDH. Furthermore, the significance of these risk factors in differentiating cases of resolution versus those that persist remains unknown.

## How can detection of DDH be improved?

Review of the current understanding of the aetiology and pathophysiology of DDH reveals evidence for both genetic [5] and environmental contributions to DDH. Hypothesised environmental risk factors may be broadly categorised as epidemiological, nutritional, hormonal and connective tissue composition (Table 1).

**Table 1:** Summary of categorised possible DDH risk factors requiring further elucidation

	Maternal	Infant
1. Epidemological	Age	Gestation
	Parity	Birth order
	Ethnicity	Gender
	Month of conception	Date, season of birth
2. Nutritional	Weight, height (BMI)	Birthweight
	Use of dietary supplements	Growth (height, weight)
	Alcohol history	Breast/bottle milk
	Smoking history	Age of weaning
Serum	Vitamin C	Vitamin C
	Vitamin D	Vitamin D
	Calcium	Calcium
3. Hormonal	Hormonal contraception use	
Serum	Relaxin	Relaxin
Breast milk	Relaxin	Prolidase
Urine	Oestrogens	Oestrogens
4. Connective tissue	Beighton score/similar joint laxity assessment	Umbilical cord collagen (content, ratio)
	Pelvic joint syndrome	

### Epidemiological

There is recognised variation in the incidence rate of DDH worldwide—ranging from 0.1 to >10 % [6]. Highest incidence populations can be found in Finland, Croatia and Canada (5–195 per 1,000), with very low incidences amongst populations in sub-Saharan Africa and Hong Kong (0–0.1 per 1,000) [7]. Geographical and cultural factors regarding climate differences and the practice of swaddling respectively may in part explain this variation [8, 9].

Within individual populations, DDH prevalence can show marked seasonal variation [10–12]. Although the trend has been most commonly observed with an inclination towards the winter months, the converse is reported in Finland with a peak in the months of June and July [13]. Current evidence lacks consistency, and as Siffel et al. [14] recommended, more robust analytical approaches such as time-series analyses are required [14]. Progress is underway: the first study to mathematically model the birth month of children with DDH was published earlier this year, demonstrating seasonal variation across all groups (both hemispheres), with peak incidences most frequently observed in mid-March and mid-October [15]. As acknowledged by the study’s authors, to make sense of these patterns of seasonal variation, we must understand their role in the context of a wider complex interplay of extrinsic physical, intrinsic physical, metabolic and genetic factors.

### Nutritional

Nutrition plays a major role in the development of canine DDH. In genetically predisposed dogs, a number of dietary excesses are believed to increase the frequency and severity of hip dysplasia. Excess energy consumption or ‘overnutrition’ is associated with rapid growth rates and relative overloading of the skeleton. This is believed to increase the frequency and severity of DDH in genetically susceptible dogs, most markedly if in the first 6 months of life [16].

The two main aetiological categories in canine hip dysplasia (CDH) have been proposed as abnormal progression of endochondral ossification and hip joint laxity causing instability [17]. Relating these principles to diet, young dogs lack a protective mechanism against excess dietary calcium [16]. High levels of calcium delay endochondral ossification and skeletal remodelling by reducing osteoclastic activity. Excess vitamin D, by increasing intestinal absorption and renal resorption of calcium, is believed to be similarly harmful in dogs genetically predisposed to DDH.

Regarding the interaction between diet and hip joint laxity, the importance of vitamin C for healthy collagen synthesis is well understood and was previously believed to be protective against canine DDH [18]. The administration of high dose dietary vitamin C in the controlled clinical setting has however failed to support this theory [19]. In addition, some studies show that excess vitamin C causes hypercalcaemia and delayed bone remodelling as described above [20].

Unlike young dogs, neonates exhibit protective parathyroid, renal and skeletal regulatory mechanisms to control calcium and vitamin D levels. To the best of our knowledge, no data have been published regarding maternal and neonatal levels of calcium, or vitamins D or C, and risk of DDH.

### Hormonal

Relaxin is a 6-kDa polypeptide pregnancy hormone that increases the secretion of collagenase and plasminogen activator—two important enzymes for collagenolysis. Two main hypotheses for the role of relaxin in DDH exist. The first describes its direct effect on foetal ligament laxity by influencing connective tissue metabolism alongside oestrogens and progesterone. Higher and more prolonged levels of relaxin have been reported in canine serum of animals with hip dysplasia [21] and in the cord blood serum of babies with hip dysplasia compared to those without hip pathology (28.7 vs. 19.8 pg/ml [22]). This however did not reach statistical significance (*p* = 0.086) in a cohort size of 24 babies, and subsequent investigation has failed to show an association between umbilical vein blood relaxin levels and neonatal hip instability [23].

A contradicting hypothesis exists to explain a higher incidence of DDH with lower relaxin concentrations. One of the main assumed functions of relaxin is relaxation of the mother’s pelvic ligaments to prepare the birth canal for parturition. A study has shown lower relaxin concentrations in the cord blood of newborns with clinical symptoms of hip instability compared to newborns with inconspicuous hip findings. Hence, an association between lower relaxin levels and poorer preparation of the pelvis has been suggested to cause higher pressures to be exerted on the foetus perinatally [24]. This indirect influence of relaxin fits with a longstanding theory that intrauterine pressure or that developed during delivery may contribute to hip instability [25, 26].

The direct effects of oestrogen and progesterone on the collagen and elastin content of the joint capsule have been investigated in animal models. These show an increased rate of hip dislocation with administration of progesterone versus a protective effect with administration of oestrogen [27].

Urine analysis has thus far shown no significant differences in oestrogen output between newborns with DDH and matched controls [28]. However a positive correlation between serum progresterone and serum relaxin concentration has been reported, with attenuation of serum relaxin concentration associated with use of hormonal contraception [29].

The ubiquity of relaxin and a lack of clear definition for its many actions make for an obvious focus of further studies. A greater understanding of relaxin receptor expression of the developing foetal hip and hence the possibility of varying susceptibilities to this hormone is required.

### Connective tissue composition

Connective tissue disorders include a spectrum of cartilage, collagen, fibrillin and matrix protein abnormalities. In 1978, Ponseti described his theory of acetabular cartilage dysplasia following histological analysis of six infants with DDH [30]. The pathological changes documented include a loss of proteoglycans and an increase in collagen deposition in the lateral acetabular roof, manifesting as a ridge of degenerating acetabular cartilage—detected clinically as a positive Ortolani sign.

Systemically, recognition of associated joint hypermobility and of inguinal hernias in children with DDH has directed the focus of a number of studies to identify differences in collagen content, distribution and metabolism of the joint capsule and ligaments [31–34]. The specific appearance of hernias abnormally early in life favours the hypothesised role of relaxin exerting its effects on collagenase in these infants.

Prolidase is a cytosolic exopeptidase that plays a crucial role in collagen degradation and in the recycling of proline. Increased serum prolidase activity in patients with DDH suggests increased collagen turnover, with a positive correlation reported between prolidase level and disease severity [35]. Soran et al. [35] compared 36 DDH patients to 33 controls, all between the ages of 9 and 18 months. Larger population studies, to also include a wider age range, are required to show consistency and add power to these findings. It is as yet unclear whether and how prolidase activity may be a useful marker in differentiating between cases of DDH likely to resolve or persist.

## Risk stratification: baby steps to refined screening

Our review of the most up-to-date evidence highlights the need for better characterisation of the natural history of human DDH. Efforts to improve detection of DDH have been based on identification of infants at increased risk. Neither the prenatal and postnatal factors that predispose to hip instability nor the determinants of its resolution or persistence are well understood.

A number of epidemiological, nutritional, hormonal and connective tissue influences on ligament laxity have been hypothesised. Evidence for these potential risk factors is however provided largely by canine studies, with a paucity of consistent or strong evidence in humans. Specifically, we lack large prospective studies that capture the breadth of the environmental risk factors hypothesised. Such studies would provide data to aid the design of further focussed, high-quality human studies of specific, potentially modifiable maternal, pre- and antenatal risk factors for DDH. In turn, we may progress towards a clearer understanding of the origins of early degenerative hip disease, using robust evidence to best direct screening and surgical resources in the future.
